# Increasing prevalence of malaria and acute dengue virus coinfection in Africa: a meta-analysis and meta-regression of cross-sectional studies

**DOI:** 10.1186/s12936-023-04723-y

**Published:** 2023-10-06

**Authors:** Tewelde T. Gebremariam, Henk D. F. H. Schallig, Zeleke M. Kurmane, Jonas B. Danquah

**Affiliations:** 1 School of Graduate Studies and Research, Frantz Fanon University, Hargeisa, Somaliland; 2 Department of Medical Microbiology, Academic Medical Centre, University of Amsterdam, Amsterdam, The Netherlands; 3 School of Medical Laboratory, Institute of Health, Jimma University, Jimma, Ethiopia; 4 Animal Research Institute, Animal Health Division, Accra, Ghana

**Keywords:** Prevalence, Malaria, Dengue fever, Dengue virus, *Plasmodium falciparum*, Coinfection, Meta-analysis, Meta-regression

## Abstract

**Background:**

Malaria and dengue fever are the leading causes of acute, undifferentiated febrile illness. In Africa, misdiagnosis of dengue fever as malaria is a common scenario. Through a systematic review of the published literature, this study seeks to estimate the prevalence of dengue and malaria coinfection among acute undifferentiated febrile diseases in Africa.

**Methods:**

Relevant publications were systematically searched in the PubMed, Cochrane Library, and Google Scholar until May 19, 2023. A random-effects meta-analysis and meta-regression were used to summarize and examine the prevalence estimates.

**Results:**

Twenty-two studies with 22,803 acute undifferentiated febrile patients from 10 countries in Africa were included. The meta-analysis findings revealed a pooled prevalence of malaria and dengue coinfection of 4.2%, with Central Africa having the highest rate (4.7%), followed by East Africa (2.7%) and West Africa (1.6%). Continent-wide, *Plasmodium falciparum* and acute dengue virus coinfection prevalence increased significantly from 0.9% during 2008–2013 to 3.8% during 2014–2017 and to 5.5% during 2018–2021 (p = 0.0414).

**Conclusion:**

There was a high and increasing prevalence of malaria and acute dengue virus coinfection in Africa. Healthcare workers should bear in mind the possibility of dengue infection as a differential diagnosis for acute febrile illness, as well as the possibility of coexisting malaria and dengue in endemic areas. In addition, high-quality multicentre studies are required to verify the above conclusions.

*Protocol registration number*: CRD42022311301.

## Background

Acute undifferentiated febrile illness (AUFI) is one of the most frequent reasons for seeking healthcare in Africa [1]. AUFI usually begins with nonspecific symptoms such as the sudden onset of fever, which rarely progresses to prolonged duration, headaches, chills, and myalgia, which may later involve specific organs. It can range from a mild and self-limiting illness to an advancing, deadly disease [2]. Malaria and dengue fever are leading causes of AUFI [3].

Africa carries the highest global malaria burden, with 2000 million cases (92%) in 2017 alone [4]. Human malaria is mainly caused by four *Plasmodium* species, namely, *Plasmodium falciparum*, *Plasmodium vivax*, *Plasmodium malariae*, and *Plasmodium ovale*, with a variable geographic distribution. *P. falciparum* accounts for nearly all malaria deaths in sub-Saharan Africa, which bears over 90% of the global malaria burden [5]. Likewise, the prevalence of dengue in the region has dramatically increased over the past few decades, although this specific infection is neither systematically investigated nor generally considered by clinicians [6]. In 2013, approximately 16 million apparent and over 48 million inapparent cases of dengue were estimated to have occurred, and most countries on the continent reported recurrent outbreaks [7]. Dengue fever is caused by four genetically distinct dengue viruses (serotypes 1–4) [8].

Although malaria or dengue virus monoinfections can be severe, concomitant infections could be even more fatal [9, 10]. The two mosquito-borne diseases have an overlapping epidemic pattern in Africa [11]. Similar main symptoms, such as fever, headache, myalgia, arthralgia, rash, nausea, diarrhoea, vomiting, and abdominal pain, are present in both of these illnesses [12]. Due to their similar clinical presentations, possible concurrent malaria-dengue fever is often neglected [13] and generally misdiagnosed as malaria only [6, 14]. Misdiagnosis is more probable during coinfection than mono-infection, and this may result in slow identification of dengue fever outbreaks with potentially high morbidity and mortality [6, 15].

A concurrent second infection may obscure the symptoms of either infection, and the treatment regimens for these co-infection are not the same as those for mono-infections [16], hence delaying the implementation of the appropriate treatment regimen or leading to serious complications [12, 16].

The highly mobile lifestyle of the population today, the increased activities made available by reliable global transportation networks, and climate change are anticipated to enhance the prevalence of co-infection with dengue and malaria [17]. This review aimed to gather evidence to answer how common could *Plasmodium* and dengue virus coinfection in Africa be? The specific review objective was to determine the prevalence of malaria and acute dengue coinfection in Africa (by region and study time period).

## Methods

The protocol of the review was registered in the International Prospective Register of Systematic Reviews, PROSPERO (CRD42022311301), and followed the Preferred Reporting Items for Systematic Reviews and Meta-Analyses (PRISMA) checklist [18]. The Joanna Briggs Institute (JBI) Critical Appraisal Checklist for Studies Reporting Prevalence Data [19] was used to assess the methodological quality of the included studies.

### Inclusion and exclusion criteria

Cross-sectional studies that reported *Plasmodium* and dengue virus coinfection among uncomplicated febrile cases attending health facilities in African regions were included. According to the United Nations, Africa is divided into five regions: Northern Africa, Central or Middle Africa, Southern Africa, East Africa, and Western Africa [20]. Similarly, the World Bank lists a total of 48 countries in the sub-Saharan African region [21].

Malaria might be diagnosed by malaria rapid diagnostic tests, microscopy and/or polymerase chain reaction, while dengue fever might be identified through an antigen or antibody test and/or reverse transcriptase-polymerase chain reaction. Acute dengue or dengue fever was defined as positive for dengue IgM or NS1 antigen testing or RT‒PCR.

Reviews, grey literature, books, posters, conference proceedings, unpublished articles, articles whose full texts could not be obtained or were not available in English or that reported asymptomatic infections, studies of malaria without coinfection, reports of dengue without coinfection, case–control studies, experimental studies, reports of coinfection in malaria patients, reports of coinfection in dengue patients, and studies outside Africa were excluded. The primary outcome measure was the prevalence of malaria and dengue coinfections in Africa.

### Databases and search strategy

The CoCoPop mnemonic (condition, context, and population) [22] was used to formulate the review question and systematically search all relevant studies from PubMed, Cochrane Library, and Google Scholar databases until 19 May 2023. The search strategy used was as follows:

### PUBMED

((("Malaria, Falciparum"[Mesh] OR "Malaria"[Mesh] OR "Plasmodium"[Mesh] OR "Plasmodium falciparum"[Mesh] OR malaria [tiab] OR falciparum [tiab] OR marsh fever [tiab] OR plasmodium [tiab] OR plasmodium falciparum [tiab]) AND ("Dengue"[Mesh] OR "Dengue Virus"[Mesh] OR dengue [tiab] OR dengue fever [tiab] OR breakbone fever [tiab] OR break bone fever [tiab] OR dengue virus*[tiab])) OR ((malaria [tiab] OR falciparum [tiab] OR plasmodium [tiab] OR marsh fever [tiab] OR plasmodium falciparum [tiab]) AND (dengue [tiab] OR dengue fever [tiab] OR breakbone fever [tiab] OR break bone fever[tiab] OR dengue virus*[tiab]) NOT MEDLINE[sb])) NOT systematic [sb].

## Cochrane Library

**Table:** 

ID	Search
#1	MeSH descriptor: [Malaria] explode all trees
#2	MeSH descriptor: [Plasmodium] explode all trees
#3	(malaria):ti,ab,kw OR (plasmodium):ti,ab,kw (Word variations have been searched)
#4	MeSH descriptor: [Dengue] explode all trees
#5	(dengue):ti,ab,kw OR (dengue fever):ti,ab,kw (Word variations have been searched)
#6	#1 OR #2 OR #3
#7	#4 OR #5
#8	#6 AND #7

### Study quality appraisal and data extraction

The Joanna Briggs Institute System for the Unified Management, Assessment, and Review of Information (JBI SUMARI) tool [23] was used to screen each article and extract relevant data for the review. Two of the authors (TT and JD) independently screened each article at the abstract and full-text levels. The discrepancy between the two reviewers was resolved through discussion. Articles endorsed in the full-text screening were subjected to the JBI critical appraisal tool. Those with good quality scores were subjected to data extraction. Data extraction included the first author’s last name, publication year, country/region of study, sample size, number of malaria and dengue coinfections, and demographic characteristics (age, gender) of patients with coinfections. The JBI criteria were used to score the quality of each study. Studies with a score greater than or equal to four were considered to have sufficient quality to be included in the meta-analysis.

### Statistical analysis

The random-effect models was used to determine the prevalence estimates and their 95% confidence intervals (CIs). The I^2^ statistic and Cochran’s Q-test were used to measure the heterogeneity of the included studies, and meta-regression analysis was used to investigate the factors associated with heterogeneities in stratified meta-analyses. The publication bias was evaluated using Begg and Mezumdar rank correlation tests and assessed the relationship between malaria prevalence and dengue fever prevalence using spearman correlation. A subgroup analysis was performed to determine the prevalence by study time period and region and calculated the odds ratio (OR) and 95% CI of prevalence to estimate the effect of age and gender. A p value < 0.05 was considered to indicate statistical significance. The data was analysed using Comprehensive Meta-analysis (Version 3) software.

### Patient and public involvement

This study was performed without patient or public involvement.

## Results

### Literature retrieval and characteristics of the included studies

**Fig. 1:**
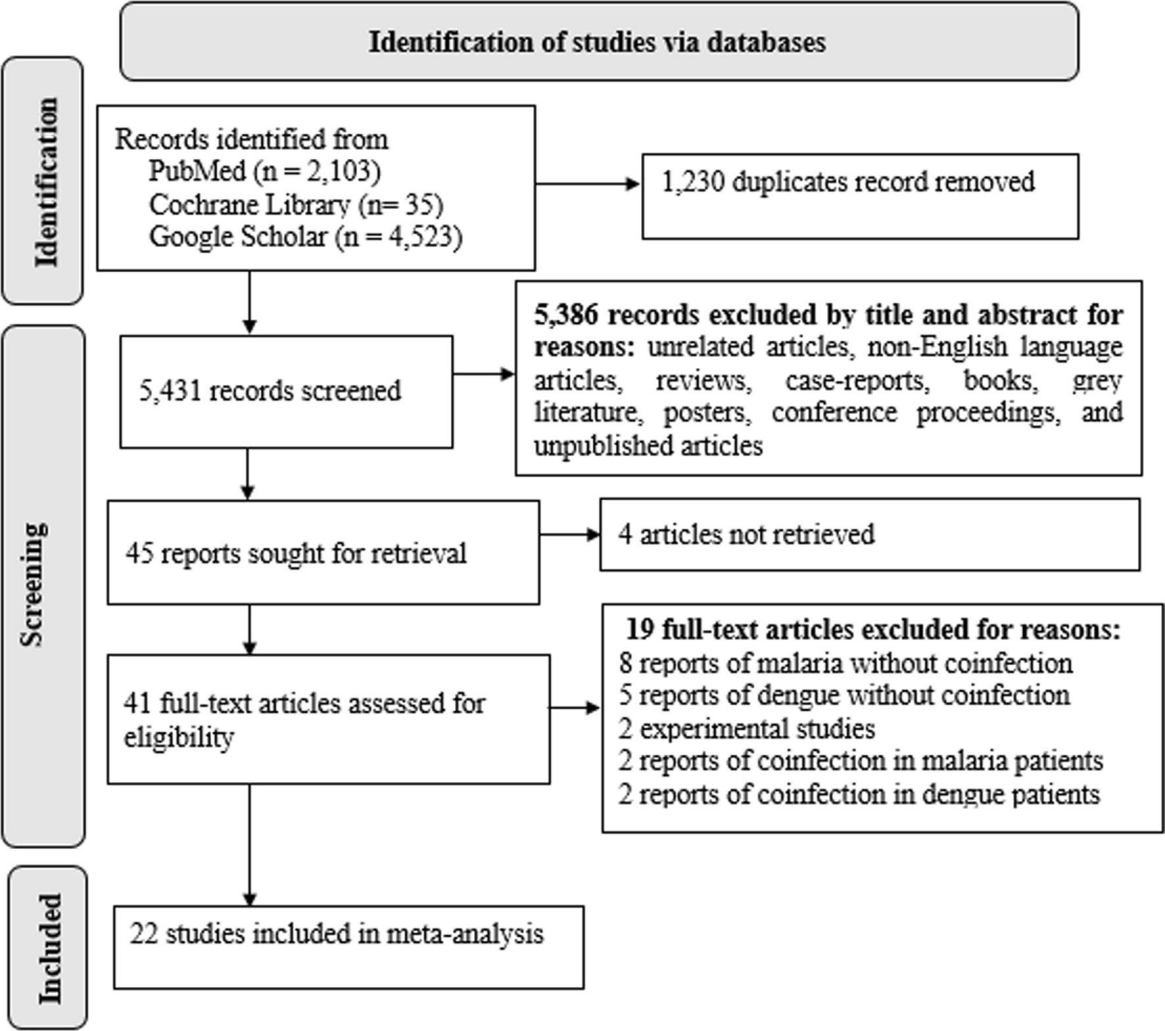
Flow diagram for study screening and selection process

The article screening and selection process is depicted in the PRISMA flowchart (Fig. 1). A total of 6661 records were identified during literature retrieval from the databases, and of those, 5431 had their titles and abstracts screened. The full texts of 22 studies involving 22,803 patients with AUFI were included in the quantitative synthesis [24–44] (Table 1). While 14 studies [29, 30, 33–44] were carried out in or after 2015, eight [24–28, 31, 32, 45] of the included studies were conducted prior to that year. Nine of the included studies were carried out in Nigeria [24, 26, 27, 29, 30, 32, 35, 36, 38] and five in Cameroon [33, 34, 41, 42, 44], and the remaining eight studies were in Tanzania [25, 39], Kenya [31, 40], Senegal [28], Sierra Leone [45], Ethiopia [43], and the DRC [37]. All the included studies used a cross-sectional design and included patients with AUFI.

**Table 1:** Characteristics of the included studies

No	Author, year	Ref	Country (Study period)	Age range^**±**^ or IQR^**#**^ (years)	Mean^**£**^ or median^*^ age (years)	Gender (M/F)	Sample (n)	Malaria mono-infection	Malaria detection method	DENV mono-infection	Dengue detection method	Coinfection	JBI Checklist Score
1	Sow 2016	[28]	Senegal (2009–2013)	1–90^±^	13^*^	0.25	13,845	7386	Microscopy, RDT	2	ELISA, PCR	1	7
2	Abdulaziz 2020	[38]	Nigeria (2017)	13–36^#^	24^*^	0.65	424	81	RDT	332	ELISA	67	5
3	Ayorinde 2016	[27]	Nigeria (2014)	3–70^±^		0.36	60	24	Microscopy, PCR	0	ELISA	1	4
4	Baba 2013	[24]	Nigeria (2008)	< 1–80^±^	32^£^	0.82	310	2	Microscopy	53	PRNT	4	6
5	Dariano 2017	[45]	Sierra Leone (2012–2013)	6–45 + ^±^	NS	NS	1260	291	RDT	40	ELISA, RDT	7	6
6	Miri 2021	[30]	Nigeria (2019)	0–51 + ^±^	29.9^£^	0.81	94	11	PCR	52	ELISA	5	4
7	Oyero 2014	[26]	Nigeria (2013)	4–82^±^	31^£^	0.77	188	25	NS	48	ELISA	19	4
8	Onyedibe 2018	[32]	Nigeria (2014)	0–58 + ^±^	21.4^£^	0.49	529	110	Microscopy	5	ELISA	7	6
9	Nassar 2019	[35]	Nigeria (2015–2016)	0–55^±^	27.2^£^	1.18	170	71	RDT, Microscopy	2	ELISA	1	6
10	Kolawole 2017	[29]	Nigeria (2016)	0–70^±^	NS	0.85	176	NS	RDT	90	ELISA, PCR	5	5
11	Ali 2020	[39]	Tanzania (2015)	1–70^±^	22^*^	0.71	149	5	PCR	7	PCR	2	5
12	Shah 2020	[40]	Kenya (2017)	1–17^±^	6.7^£^	1.19	1022	291	Microscopy	211	ELISA, PCR	150	5
13	Akelew 2022	[43]	Ethiopia (2019–2020)	20–35^#^	35^*^	1.30	200	22	RDT, Microscopy	11	ELISA, PCR	4	6
14	Chipwaza 2014	[25]	Tanzania (2013)	2–13^±^	NS	1.04	364	83	Microscopy	29	ELISA, PCR	18	6
15	Obonyo 2018	[31]	Kenya (2011)	1–82^±^	20^*^	NS	1332	174	Microscopy	23	PCR	7	4
16	Tchetgna 2021	[44]	Cameroon (2020)	0–84^±^	29^£^	0.80	320	0	RDT, microscopy	18	PCR	23	5
17	Galani 2020	[41]	Cameroon (2019–2020)	0.58–80^±^	23.2^£^	1.05	174	122	RDT, microscopy	0	ELISA	12	6
18	Nkenfou 2021	[42]	Cameroon (2015)	0.5–15^±^	3.2^£^	0.88	349	115	Microscopy	70	RDT, ELISA	68	4
19	Proesmans 2019	[37]	DRC (2015–2016)	2–68^±^	21^£^	0.87	342	149	Microscopy, RDT	13	PRNT, PCR, ELISA	6	6
20	Tchuandom 2018	[33]	Cameroon (2016–2017)	0–15^±^	7.1^£^	1.06	961	350	RDT	98	ELISA	40	4
21	Yousseu 2018	[34]	Cameroon (2017)	0.25–81^±^	26^*^	0.58	114	19	RDT	8	PCR	3	5
22	Otu 2019	[36]	Nigeria (2017)	1–99^±^	34^£^	0.60	420	17	Microscopy	8	LFIA	7	6

*Median age, *DRC* Democratic Republic of Kongo, *ELSIA* Enzyme-linked Immunosorbent Assay, *LFIA* Lateral Flow Immunoassay, *JBI* Joanna Briggs Institute, *PCR* Polymerase Chain Reaction, *PRNT* Plaque Reduction Neutralisation Test, *RDT* Rapid Diagnostic Test, *IQR* Interquartile Range^#^

The range or interquartile rage (IQR) of age of participants was reported in 20 and two of the 22 studies, while the mean or median age were stated in 14 and six studies, respectively. The mean or median age was not mentioned in two studies [29, 45]. Eighteen studies included both children and adults, while four studies [25, 33, 40, 42] exclusively focused on children. Twenty studies were performed on both men and women. The gender of the participants was not specified in the two studies [31, 45]. Six studies [28, 35, 37, 41, 43, 44] used both RDT and microscopy; six studies used only standard microscopy [24, 25, 31, 32, 40, 42]; five studies [29, 33, 34, 38, 45] used only rapid diagnostic testing (RDT); two studies [30, 39] used only PCR; and one study [27] used both microscopy and PCR for malaria diagnosis. However, the detection method was not specified in one study [26].

The included studies’ JBI checklist scores varied from four to seven (Table 1). No study received a score of nine. Inappropriate sampling techniques (n = 15) and unstandardised outcome measurements (n = 10) were the most common methodological issues in the included studies.

### Plasmodium falciparum and dengue virus coinfection

**Fig. 2:**
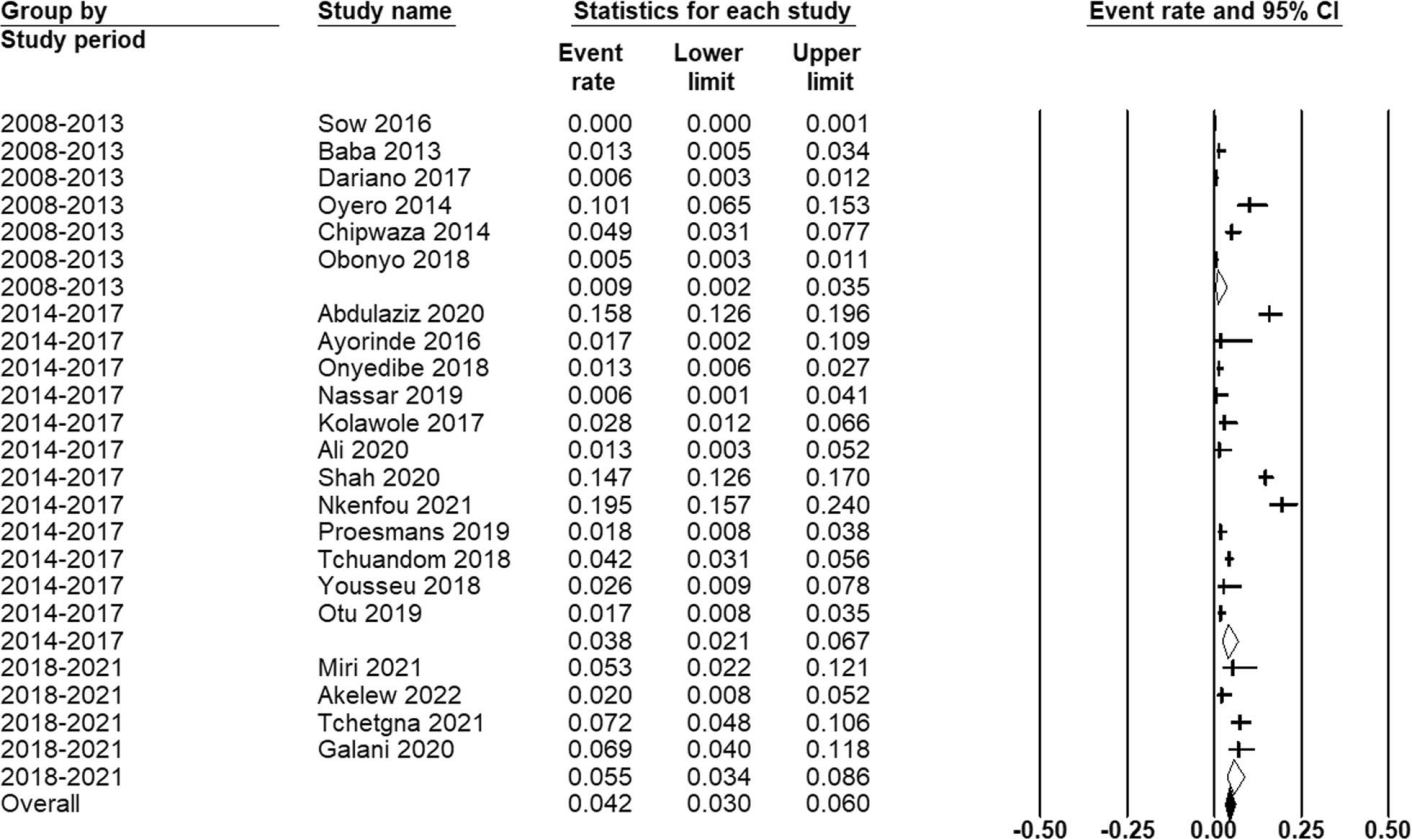
Forest plot showing the results of meta-analyses of *Plasmodium* and dengue virus coinfection among patients acute undifferentiated febrile illness during 2008–2021

**Fig. 3:**
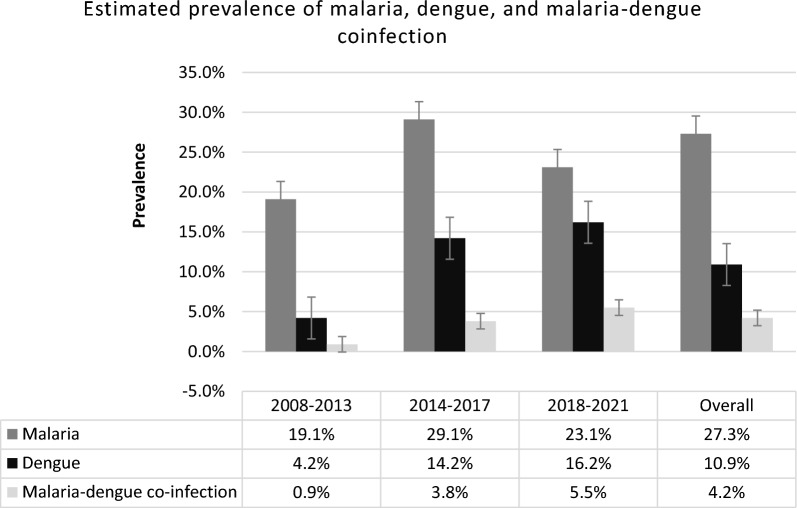
Estimated prevalence of malaria, dengue, and malaria-dengue coinfection among patients with acute undifferentiated febrile illness during 2008–2021. The error bars indicate the standard deviation of the percentages

The random-effect model estimator was used in the meta-analysis. The pooled prevalence of malaria and dengue coinfection in Africa was 42 (95% CI 30–60) per 1000 AUFI cases. This estimate substantially increased from 9 (95% CI 2–35) during 2008–2013 to 38 (95% CI 21–67) during 2014–2017 and then to 55 (95% CI 34–86) during 2018–2021 (Figs. 2 and 3). Between-study heterogeneity was found to be significantly high (I^2^ = 95.18; Q test p = 0.00), and no significant publication bias was observed (Kendall’s tau p = 0.176). The high degree of heterogeneity was significantly related to the study time period (p = 0.0414) (Table 2).

**Table 2:** Meta-regression with categorical covariates with random-effect model

Covariate	Category	B	SE	95% CI for Logit event rate		Category p-value	Covariate p-value
				Lower	Upper		
Study period	Intercept	− 4.513	0.462	− 0.452	− 0.361	0.0000	
	2008–2013	Reference					
	2014–2017	1.264	0.567	0.153	2.374	0.0258	0.0414
	2018–2021	1.562	0.718	0.155	0.2969	0.0296	
Region	Intercept	− 3.031	0.5213	− 4.053	− 2.010	0.0000	
	Central Africa	Reference					
	East Africa	− 0.539	0.816	− 2.137	1.060	0.5089	0.3336
	West Africa	− 1.031	0.696	− 2.395	0.334	0.1387	
Sample size	Intercept	− 3.607	0.410	− 4.410	− 2.804	0.0000	
	< 200	Reference					
	≥ 200	0.093	0.520	− 0.925	− 1.110	0.8586	0.8586

B, Coefficient, *SE* Standard error

**Fig. 4:**
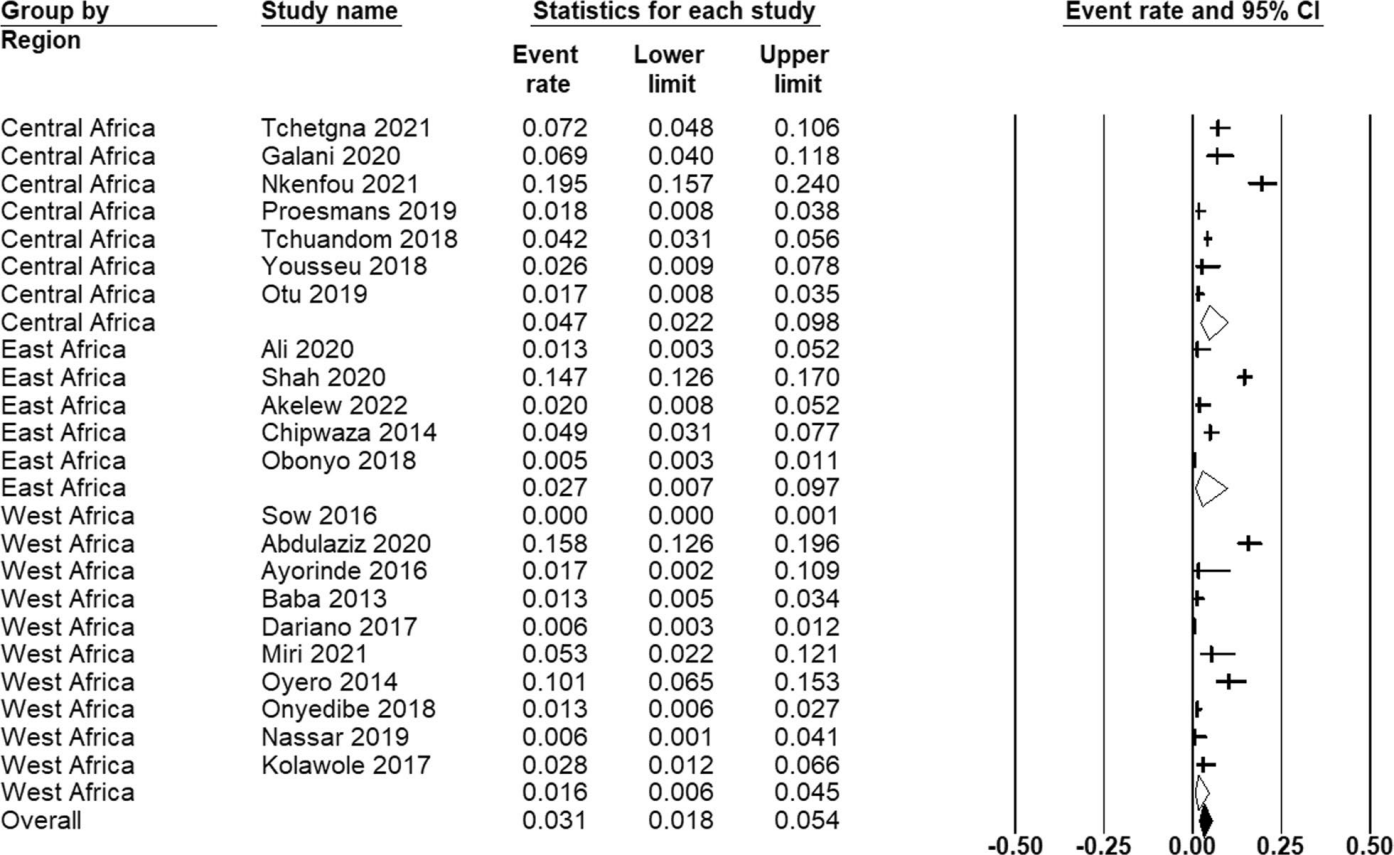
Estimated prevalence of malaria-dengue coinfection among patients with acute undifferentiated febrile illness (AUFI) across different regions in Africa

The prevalence of malaria-dengue coinfection across the three African regions ranges from 16 per 1000 febrile cases (95% CI 6–45) in West Africa to 27 (95% CI 7–97) in East Africa to 47 (95% CI 22–98) in Central Africa (Fig. 4).

The study time period was significantly related to the effect size (Table 2). In other words, the prevalence of malaria and acute dengue coinfection significantly increased over time.

**Fig. 5:**
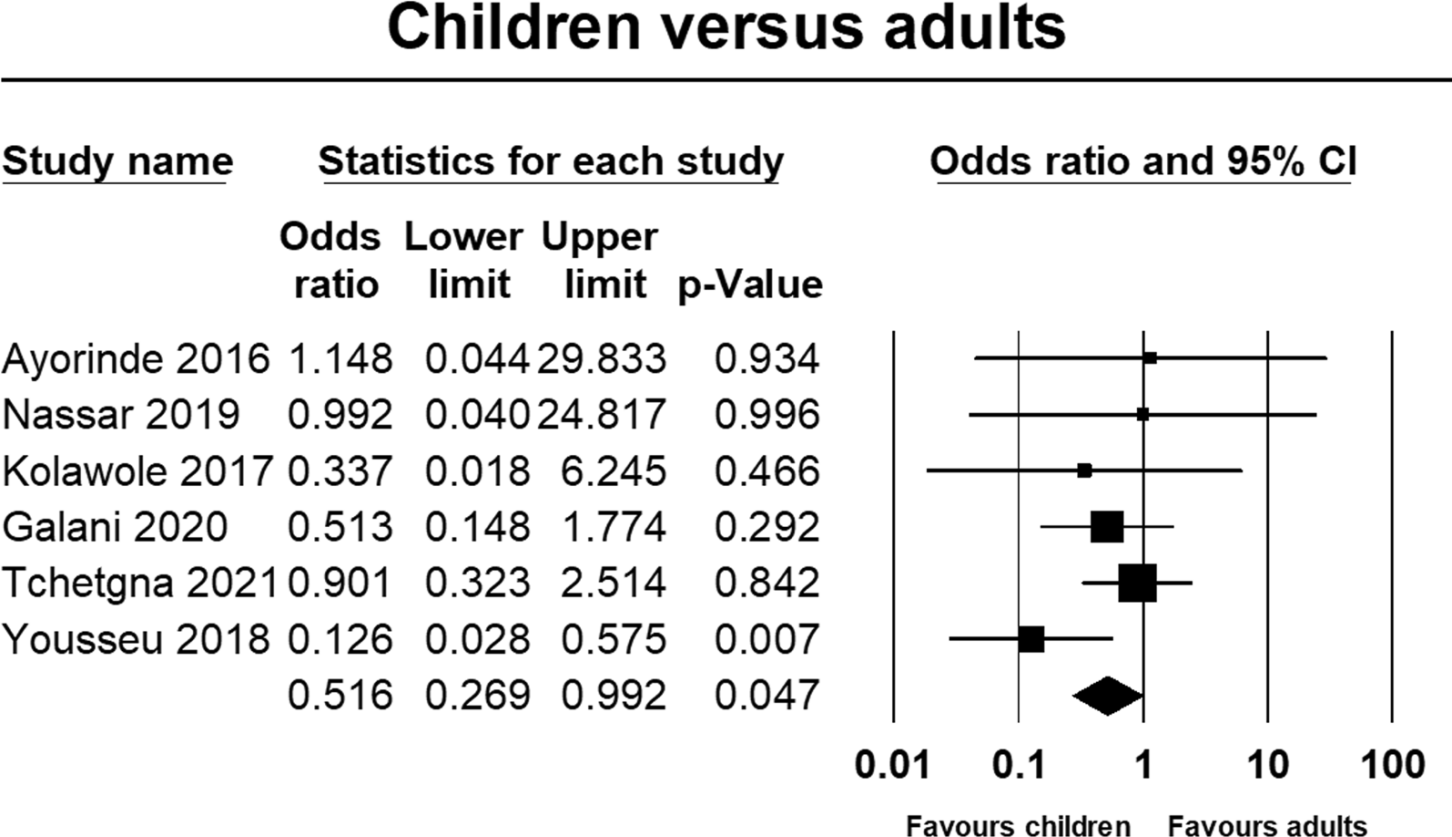
Forest plot of age difference in the prevalence of malaria and dengue coinfection

**Fig. 6:**
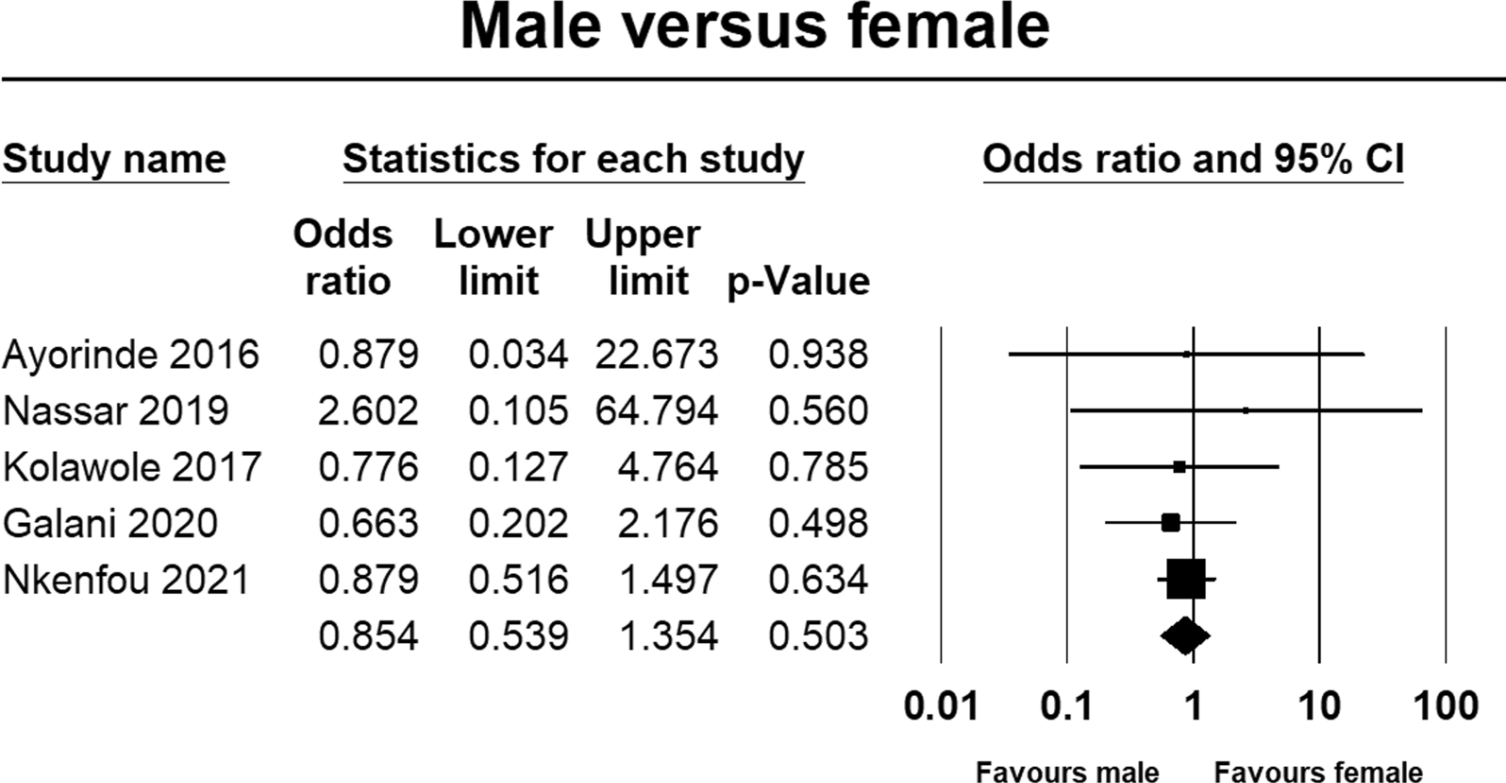
Forest plot of gender difference in the prevalence of malaria and dengue coinfection

The prevalence of *Plasmodium* and dengue virus coinfection was significantly higher in children than adults (Fig. 5; OR = 0.52, 95% CI 0.27, 0.99, p = 0.047); however, there was no statistically significant difference between males and females (Fig. 6; OR = 0.85, 95% CI 0.54, 0.135, p = 0.503).

### Association between malaria and dengue fever

**Fig. 7:**
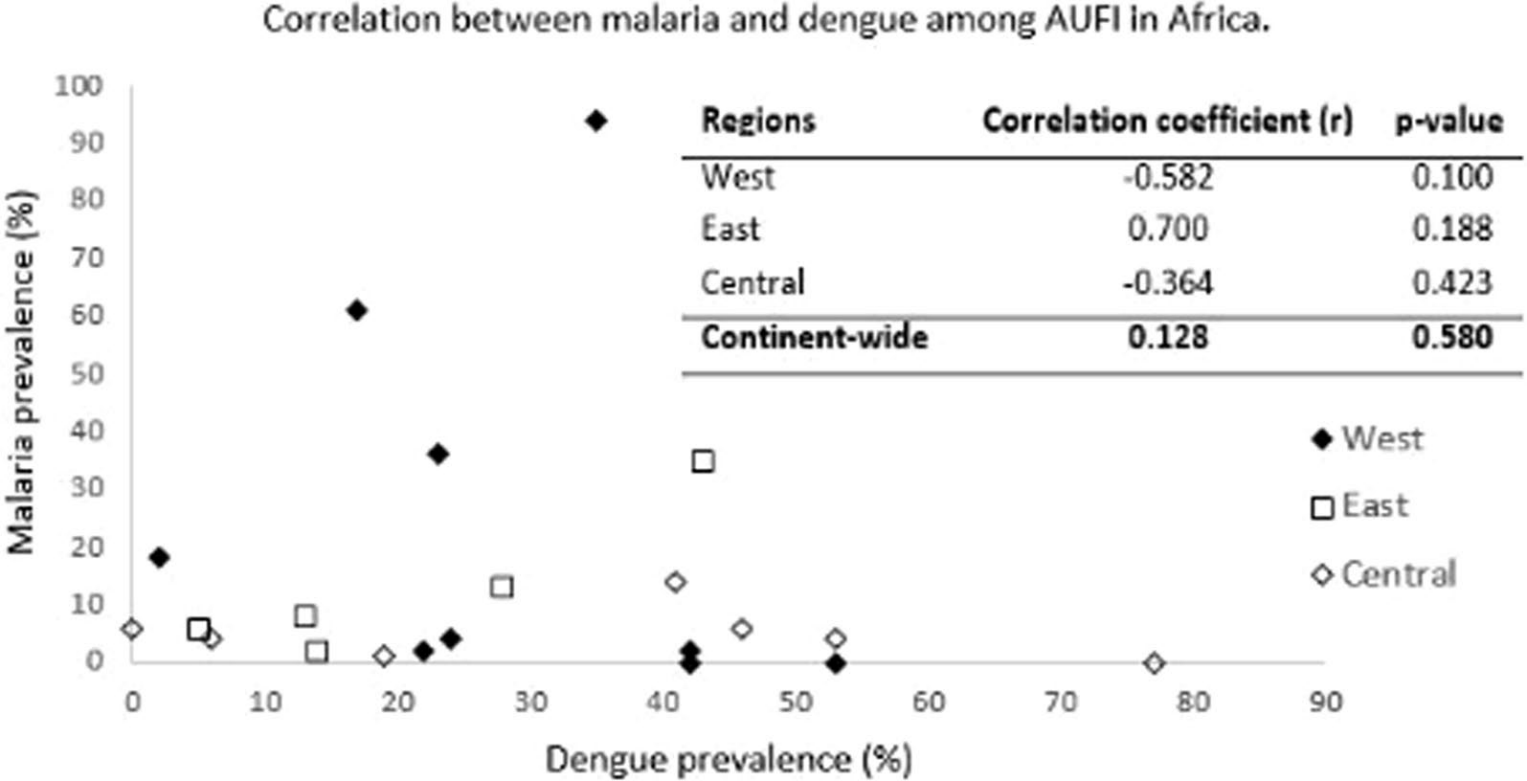
Correlation between malaria and dengue fever among acute undifferentiated febrile patients (AUFI) in three regions of Africa

A nonsignificant positive correlation (r = 0.128, p = 0.580) was observed between malaria and dengue fever prevalence among acute undifferentiated febrile illnesses (AUFI) in Africa during 2008–2021 continent-wide (Fig. 7). In all three regions, the correlation was not statistically significant. Interestingly, malaria prevalence was found to be higher than dengue in all three regions.

## Discussion

Malaria has a complicated pathophysiology, causing pathologic alterations in all bodily systems. Direct red blood cell destruction and nonspecific inflammatory and immune responses are the major mechanisms involved [46]. Similarly, dengue virus infection involves a multi-organ system and is attributed to a complex interplay between the virus, host genes, and host immune response [47]. The dengue clinical spectrum includes asymptomatic infection, mild febrile sickness (dengue fever), and more severe presentations, including dengue shock syndrome and dengue haemorrhagic fever [48]. Clinical presentations of malaria and dengue are similar, with minor differences. For instance, malaria can be chronic, while dengue cannot. In addition, atypical lymphocytosis, haemoconcentration, and thrombocytopenia are strong predictors of dengue, whereas anaemia is a major symptom seen in malaria infections, which is a consequence of the merozoites (blood stages) causing intense intravascular haemolysis [13, 16].

*Plasmodium* and dengue virus coinfection occur when both of these mosquito-borne diseases occur simultaneously in an individual, which may increase the severity and duration of one or both [16]. The first report of malaria and dengue virus coinfection in Africa was documented in 2005 [15]. About 22,803 acute undifferentiated febrile patients were included from 22 studies conducted in 8 African countries (Senegal, Nigeria, Sierra Leone, Kenya, Tanzania, Ethiopia, Cameroon, and the DRC) for approximately 13 years.

Based on the meta-analysis, the pooled prevalence of malaria and dengue fever coinfection was 4.2%, and the highest rate was recorded in Central Africa (4.7%), followed by East Africa (2.7%) and West Africa (1.6%). This result is lower than the finding of a study [49] on a meta-analysis of severe malaria and dengue coinfections, which estimated a prevalence of 32%. The variation could be due to the differences in the study population, model estimator employed, and/or geography of the primary studies included, where the analysis was focused on studies from Africa while the other study included studies from all over the globe. In addition, uncomplicated febrile cases were included in the analysis, unlike the study above, which estimated severe malaria prevalence among the coinfections.

Across the African continent, the prevalence of co-infection with *P. falciparum* and dengue virus grew significantly from 0.9% between 2008 and 2013 to 3.8% between 2014 and 2017 and 5.5% between 2018 and 2021. This could be due to increased global transportation network dynamics, population movement, and climate change [17].

In the study, children were more affected by coinfection than adults. Children are more susceptible to mosquito-borne illnesses because they are exposed to mosquito bites for longer periods during dangerous hours [50]. Moreover, malaria [51] and dengue [52] mainly affect children due to their underdeveloped specific immunity to infection [51].

Furthermore, *Plasmodium falciparum* was the only malaria parasite specified in the coinfection among the included studies, as nearly all malaria cases in Africa are caused by *P. falciparum* [5].

The study showed a high and increasing trend of malaria and dengue coinfection prevalence in many parts of Africa. Nevertheless, healthcare workers misdiagnose dengue or malaria-dengue as malaria alone due to the institutionalisation of malaria as the primary febrile illness in the region by international development organizations and national malaria control programs [53], limited access to medical care and laboratory diagnostic facilities, a lack of awareness of healthcare workers towards non-malarial febrile illnesses [54], and the overlap of signs and symptoms of dengue with malaria [55]. Clinical misdiagnosis often leads to overuse or misuse of antimicrobials, which often accelerates the emergence and spread of antimicrobial drug resistance [56, 57]. On top of that, it causes mismanagement of the patient and dengue outbreaks [55]. Hence, the study calls for devising a standardised protocol for the diagnosis and treatment of patients with AUFIs, including dengue. In addition, healthcare professionals should always keep malaria and dengue infections in mind when dealing with such clinical presentations.

The study had some strengths and limitations. A large sample size, good-quality included studies, no evidence of publication bias among the included studies, and subgroup analysis were some of the strengths of the study; however, restriction to those published in English only, including single-centred facility-based studies, a small sample size in six studies [27, 29, 30, 34, 35, 39], and evidence of significant heterogeneity among the studies were some of the drawbacks of the study.

## Conclusion

In general, a high prevalence of malaria and dengue virus coinfection among acute undifferentiated febrile patients was found in Africa, with variable rates across regions. Children were more affected by the coinfection than adults. Healthcare workers should bear in mind the possibility of dengue infection as one of the differential diagnoses for acute febrile illness, as well as the possibility of coexisting malaria and dengue in endemic areas. In addition, high-quality multicentre studies are required to verify the above conclusions and gain more insights into malaria and dengue virus coinfection on the continent.

## Data Availability

The study data are available on reasonable request. Interested researchers should contact the corresponding author using the email provided.
