# Correction: Increasing prevalence of malaria and acute dengue virus coinfection in Africa: a meta-analysis and meta-regression of cross-sectional studies

**DOI:** 10.1186/s12936-023-04771-4

**Published:** 2023-11-03

**Authors:** Tewelde T. Gebremariam, Henk D. F. H. Schallig, Zeleke M. Kurmane, Jonas B. Danquah

**Affiliations:** 1 School of Graduate Studies and Research, Frantz Fanon University, Hargeisa, Somaliland; 2 Department of Medical Microbiology, Academic Medical Centre, University of Amsterdam, Amsterdam, The Netherlands; 3 School of Medical Laboratory, Institute of Health, Jimma University, Jimma, Ethiopia; 4 Animal Research Institute, Animal Health Division, Accra, Ghana

**Correction: Malaria Journal (2023) 22:300** **https://doi.org/10.1186/s12936-023-04723-y**

Following publication of the original article [1], it was brought to the authors’ attention that one of the names in the author list had been incorrectly spelled: ‘Henk D. F. H. Schalling’ had been written in place of ‘Henk D. F. H. Schallig’. The error has since been corrected in the original article. The authors thank you for reading this erratum and apologize for any inconvenience caused.
